# Management of intracranial undifferentiated pleomorphic sarcoma: a systematic review

**DOI:** 10.1007/s10143-025-04018-7

**Published:** 2026-01-06

**Authors:** Noah B. Drewes, Jeffrey Z. Nie, Rafal Chojak, Khizar R. Nandoliya, Rishi Jain, Harrshavasan Congivaram, Umme H. Faisal, Lara Koutah, Niyant Vora, Elek A. Wellman, Matthew W. Weber, Nathan J. Nordmann, Jeffrey W. Cozzens, Devin V. Amin, Jose A. Espinosa, Breck Jones, Bruce M. Frankel, Leslie Acakpo-Satchivi

**Affiliations:** 1 Department of Surgery, Division of Neurosurgery, Southern Illinois University School of Medicine, Springfield, IL USA; 2 Department of Neurosurgery, Neurological Institute, Cleveland Clinic Foundation, Cleveland, OH USA; 3 Department of Neurological Surgery, Feinberg School of Medicine, Northwestern University, Chicago, IL USA; 4 Neurological Surgery, Springfield Clinic, Springfield, IL USA

**Keywords:** Undifferentiated pleomorphic sarcoma, Brain tumors, Malignant fibrous histiocytoma, Systematic review, Neurosurgery, Prognosis

## Abstract

Undifferentiated pleomorphic sarcoma (UPS) is a rare and aggressive soft tissue tumor of unknown histogenesis. UPS most often arises in the extremities or trunk, where wide surgical excision is feasible. Rarely, UPS involves the intracranial space, where critical structures may inhibit complete resection. Given its rarity and historical misclassification as malignant fibrous histiocytoma (MFH), knowledge of intracranial UPS is limited, and there are no clear treatment guidelines. We systematically reviewed all available cases of intracranial UPS to consolidate existing literature and assess prognosis and management considerations. A systematic review was conducted according to PRISMA guidelines. PubMed and Embase were searched for reports of intracranial UPS or storiform-pleomorphic MFH. Eligible studies included at least one histologically confirmed case treated with surgery, chemotherapy, or radiotherapy and reported outcomes. Data on demographics, tumor location, treatment, follow-up, and survival were extracted. Event-free survival (EFS) and overall survival (OS) were analyzed by treatment modality, age, sex, and tumor location using Kaplan-Meier and log-rank tests. From 801 records, 40 studies with 48 patients were included. The median age was 45 years, and 50% were female. 15% had prior radiotherapy. Most tumors were extra-axial (45%) and nearly all patients underwent surgery, with 82.1% achieving gross total resection. Median EFS and OS were 18 and 24 months, respectively. No survival differences were seen by treatment modality, age, or sex. Overall survival was longer for extra-axial tumors compared to intra-axial or skull base tumors (*p* = 0.016). Intracranial UPS is a rare and aggressive tumor, with worse prognosis compared to those that are extracranial. Among factors that we evaluated, extra-axial location was the strongest positive prognostic factor. Surgical resection with adjuvant chemoradiation showed the longest median survival. Ongoing case reports are required to refine management guidelines and improve patient outcomes.

**Supplementary Information:**

The online version contains supplementary material available at 10.1007/s10143-025-04018-7.

## Introduction

Undifferentiated pleomorphic sarcoma (UPS) is a rare and aggressive soft tissue tumor of unknown histogenesis^,^ accounting for approximately 10% of adult soft tissue sarcomas [1–4]. It is primarily a diagnosis of exclusion based on the absence of a specific line of cellular differentiation [3, 4]. UPS typically arises in the extremities or trunk, where wide surgical excision is often feasible [5]. As a result, the added benefit of adjuvant therapies such as chemotherapy and radiation is limited [6–14]. Moreover, radiotherapy carries additional controversy due to the association with radiation-induced UPS [15]. Thus, the utility of these additional modalities for the general treatment of UPS is unclear.

The Fédération Nationale des Centres de Lutte Contre le Cancer (FNCLCC) system classifies UPS as a Grade 3 (high-grade) sarcoma based on differentiation, mitotic activity, and necrosis [16]. Consistent with this, the National Comprehensive Cancer Network (NCCN) guidelines recommend multimodal therapy, including surgical resection, radiotherapy, and chemotherapy, for all high-grade sarcomas [17]. These treatment paradigms provide context for adjuvant therapy in UPS arising in complex locations.

When UPS occurs intracranially, the high density of critical neuroanatomical structures may pose a barrier to complete resection, justifying adjuvant therapies to optimize local control [18]. Nevertheless, little is known about intracranial UPS, as studies are limited to case reports and small case series, and is further complicated by historical misclassification of malignant fibrous histiocytoma (MFH) without histologic confirmation. In this study, we systematically reviewed the literature reporting UPS involving the intracranial space. By consolidating the existing literature, we aim to summarize the current knowledge of the prognosis and management considerations of UPS.

## Methods

### Study design

A systematic review was performed according to the PRISMA guidelines (Supplementary Data 1) [19]. No formal review protocol was registered or prepared. This review was designed using the PICO framework. The population included patients with primary intracranial UPS or storiform-pleomorphic MFH. Interventions consisted of surgical resection with or without adjuvant chemotherapy and/or radiotherapy. Comparisons were made between patients receiving all three therapies, including chemotherapy, radiation, and surgery (CRS) and those receiving fewer treatments (less than CRS), as well as by age, sex, and tumor location. Other treatment combinations, such as radiation and surgery (RS), chemotherapy and surgery (CS), and surgery alone (S) were explored. The primary outcomes measured were event-free survival (EFS) and overall survival (OS). EFS was defined as the time after starting the primary treatment where the patient is free from a recurrence. OS was defined as the time after starting the primary treatment where the patient is alive.

### Search strategy

PubMed and Embase were searched on August 4, 2025, for journal articles using the following keywords and phrases: malignant fibrous histiocytoma, undifferentiated pleomorphic sarcoma, intracranial, skull, central nervous system, brain, and meninges. Full search strategies for PubMed and Embase are provided in Supplementary Data 2. Two reviewers (N.D. and J.N.) independently screened all articles for inclusion and extracted relevant data. Discrepancies were resolved through discussion with a third reviewer (R.C.) when necessary. All available studies from database inception through August 4, 2025 were eligible for inclusion.

### Eligibility criteria

Historically, UPS was classified under the broader and now outdated category of MFH, which included multiple subtypes [4, 20, 21]. However, advanced analysis of MFH have led to the recategorization or removal from the WHO sarcoma classifications [4, 20, 22]. Presently, only the storiform-pleomorphic subtype of MFH (SP-MFH) is considered an alternative label for UPS [20].

To be considered for inclusion, articles must report at least 1 patient with a diagnosis of storiform-pleomorphic MFH or UPS. Histological descriptions of a “storiform,” “cartwheel,” or “whorl” pattern were sufficient if no MFH subtype was explicitly reported. Articles must include patients treated with any combination of surgery, chemotherapy, or radiotherapy and must report exact follow-up times and outcomes. Articles describing patients with perioperative deaths were excluded, as these patients could not be evaluated for recurrence or survival outcomes secondary to their tumor. Moreover, there must be a single tumor involving the intracranial space. Articles reporting cases with multiple discrete tumors present at the time of treatment or intracranial metastases from distant sites were excluded. Conference abstracts, non-English articles, and review articles without unique patients were also excluded.

### Data extraction

From articles meeting the inclusion criteria, patient demographic information, any prior history of radiation, location of the intracranial tumor, treatment modalities used, follow-up times, and outcomes were extracted.

### Outcomes

EFS and OS were evaluated between those that received all three modalities (CRS) as their primary treatment and those that received less than CRS. Additional groups were based on divisions in age, sex, and location. Age groups were defined as pediatric (≤ 17 years) and adult (≥ 18 years). Location was divided on whether the mass was extra-axial (meninges or calvarium, outside the brain parenchyma) or intra-axial (arising within the brain parenchyma). For intra-axial tumors, anatomic subgroups included supratentorial, infratentorial, and skull base.

### Risk of bias assessment

Risk of bias was evaluated using the Joanna Briggs Institute (JBI) critical appraisal checklists for case reports and case series [23]. Each study was rated across all checklist domains, and results are in Supplementary Data 3 and 4. This appraisal demonstrated that several included case reports and case series lacked documentation of adverse events.

### Statistical analysis

Kaplan-Meier survival analysis was done to generate EFS and OS for the total cohort and subgroups with 95% confidence intervals (CIs). When the median was not reached (NR), the lower 95% bound was reported. Comparisons between treatment (CRS vs. less than CRS), age (≤ 17 years vs. ≥18 years), sex, and tumor location (extra-axial vs. intra-axial/skull base) were performed using the log-rank test, with statistical significance defined as *p* < 0.05. Reverse Kaplan-Meier survival analysis was done to calculate median follow-up with 95% CIs. All analyses were conducted using R (version 4.1.2) with the survival and survminer packages.

## Results

### Search results

**Fig. 1:**
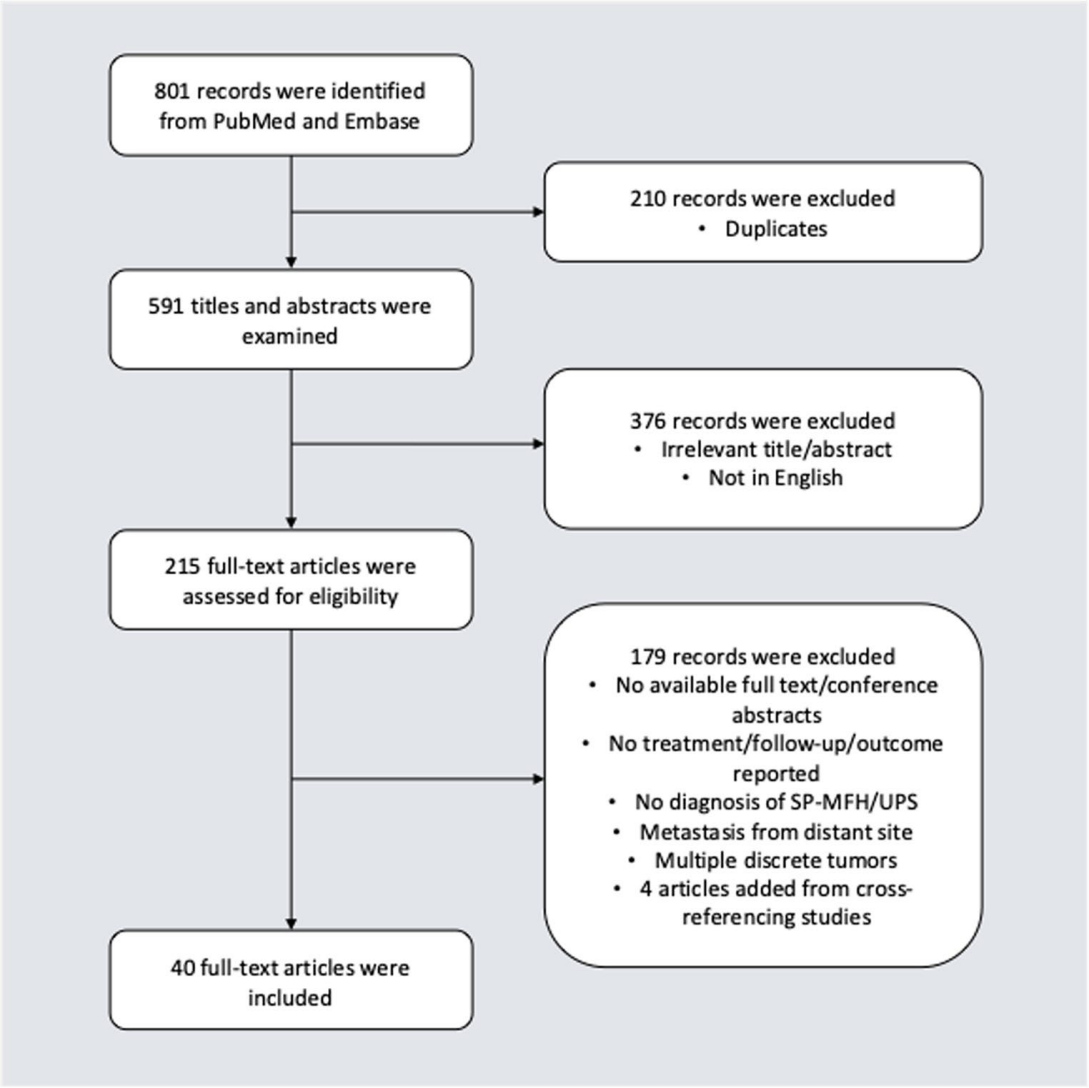
PRISMA flowchart demonstrating study selection. SP-MFH storiform-pleomorphic malignant fibrous histiocytoma, UPS undifferentiated pleomorphic sarcoma

The PubMed and Embase searches returned a total of 801 records (Fig. 1). Of these, 210 duplicates were removed, leaving 591 records. Afterwards, the titles and abstracts were screened, leaving 215 records. The full texts were subsequently reviewed and cross-referenced for additional eligible studies, leaving 40 articles for inclusion, 4 of which were added after cross-referencing [18, 24–62]. Articles were frequently excluded due to irrelevant focus, absence of follow-up or outcome of treatment, no clear diagnosis of the storiform-pleomorphic subtype of MFH, intracranial metastasis from a distant site, or a combination of the four. A comprehensive list of the literature included in this study is in Supplementary Data 5.

### Patient characteristics

From the 40 included studies, 48 patients had follow-up times and outcomes and were included for the pooled analysis. All patient demographics, surgical characteristics, and outcome data are summarized in Table 1. Overall, the median and mean ages were 45 years (IQR 18–63) and 42.5 ± 23.7 years, respectively, and 24 (50.0%) were female. Seven (14.6%) patients had a history of radiotherapy at the site of UPS, ranging from 3 to 20 years prior. Of the studies documenting initial symptoms, the most common symptom reported by patients was headaches (18/43, 41.9%), followed by a scalp mass (11/43, 25.6%) and visual disturbances (10/43, 23.3%). Of the studies reporting a specific tumor location, most tumors were extra-axial, including the meninges and calvarium (21/47, 44.7%), followed by intra-axial, including supratentorial (16/47, 34.0%), along the skull base (8/47, 17.0%), and infratentorial (2/47, 4.3%).

**Table 1:** Summary of the current literature

		*n* = 48	%
Age	Mean	42.5 ± 23.7	
	Median	45 (IQR 18–63)	
	Range	5 yrs − 75 yrs	
Sex	Male	24	50.0%
	Female	24	50.0%
		(*n* = 47)	
Location	Extra-axial (calvarium/meninges)	21	44.7%
	Intra-axial, supratentorial	16	34.0%
	Intra-axial, infratentorial	2	4.3%
	Skull base	8	17.0%
		(*n* = 43)	
Reported Symptoms	Headache	18	41.9%
	Scalp mass	11	25.6%
	Visual disturbance	10	23.3%
	Paresis/plegia	8	18.6%
	Aphasia	6	14.0%
	Altered consciousness/drowsiness/lethargy	4	9.3%
	Seizure	4	9.3%
	Ataxia/gait disturbance	3	7.0%
	Personality change	3	7.0%
	Numbness/paresthesias	2	4.7%
	Asymptomatic	1	2.3%
Primary vs. secondary	Primary	41	85.4%
	Secondary	7	14.6%
Prior RT		7	14.6%
Treatment	R	1	2.1%
	S	11	22.9%
	C + S	4	8.3%
	R + S	19	39.6%
	C + R + S	13	27.1%
Outcomes	Recurrence	22	45.8%
	Alive at time of case report	28	58.3%
	Death from disease	15	31.3%
	Death from other causes	5	10.4%
	Median OS (overall)	24 [11.0-NR]	
	Median EFS (overall)	18 [8.0-NR]	

*OS* overall survival, *EFS* event-free survival, *R* radiotherapy, *S* surgery, *C* chemotherapy

### Treatment characteristics

Patients most frequently underwent surgical resection for their UPS (47/48, 97.9%), with one (1/48, 2.1%) receiving only radiotherapy. In addition, 4 of these 48 patients (8.3%) underwent surgery and received adjuvant chemotherapy, 19 (39.6%) received adjuvant radiotherapy, 13 (27.1%) received adjuvant chemo- and radiotherapy, and 11 (22.9%) underwent surgery alone. Of the 47 patients who underwent surgery, extent of resection was reported for 39 patients. Gross total resection (GTR) was reported in 32 (82.1%) and subtotal resection (SR) was reported in 7 (17.9%).

### Follow-up and survival outcomes

Of all 48 patients, 22 (45.8%) experienced at least 1 recurrence of their UPS, of which 20 had an exact time from primary treatment to report of the first recurrence. The median follow-up time was 18 months (95% CI, 12–27). There were 20 (41.7%) total mortalities at the last recorded follow-up.

The estimated median EFS and OS of all patients were 18 months (95% CI, > 8) and 24 months (95% CI, > 11), respectively. Furthermore, the estimated median EFS for the CRS and less than CRS groups were 18 months (95% CI, > 3) and 18 months (95% CI, > 8), respectively. The estimated median OS for the CRS and less than CRS groups were 26 months (95% CI, > 12) and 22 months (95% CI, > 8), respectively. There were no significant differences in EFS (*p* = 0.279) and OS (*p* = 0.637) between these two groups. Additionally, there were no significant differences based on age (EFS *p* = 0.28, OS *p* = 0.26) or sex (EFS *p* = 0.1, OS *p* = 0.93). OS differed by location (*p* = 0.016), with the extra-axial median OS not reached (95% CI, > 22) and the intra-axial/skull base median OS being 8 months (95% CI 7–NR). The EFS for location did not significantly differ (*p* = 0.538). These statistics are summarized in Table 2.

**Table 2:** Prognostic factors for overall and event-free survival in primary intracranial UPS

		MedianOS			MedianEFS	
	N = 48		p value	N = 46		p value
Age (years)			0.279			0.328
≤17	11	26 [15-NR]		9	18[3-NR]	
≥18	37	22 [7-NR]		34	NR[>8]	
Sex			0.221			0.73
Male	24	15 [7-NR]		23	18[8-NR]	
Female	24	24 [22-NR]		23	24[3-NR]	
Location	N=47)		0.016			0.538
Extra-axial (calvarium/meninges)	21	NR [>22]		21	NR[>3]	
Intra-axial and skullbase	26	8 [7-NR]		25	11[8-NR]	
Treatment			0.637			0.279
Less than CRS	35	22[8-NR]		35	18[8-NR]	
CRS	13	26 [12-NR]		11	18[3-NR]	

*OS* overall survival, *EFS* event-free survival, *CRS* chemotherapy, radiation, and surgery

Table 3 summarizes survival by specific treatment modality. CRS had the highest median OS of 26 months (95% CI, > 12) and median EFS of 18 months (95% CI, > 3). RS showed a slightly lower OS of 24 months (95% CI, > 6). CS had a shorter median OS of 11 months (95% CI, > 4) and an EFS of 3 months (95% CI, > 1.5).

**Table 3:** Overall and event-free survival by treatment modality

	S (*n* = 11)	CS (*n* = 4)	RS (*n* = 19)	CRS (*n* = 13)
OS	NR [> 22]	11 [4-NR]	24 [6-NR]	26 [12-NR]
EFS	NR [> 3]	3 [1.5-NR]	NR [> 11]	18 [3-NR]

*OS* overall survival, *EFS* event-free survival, *R* radiotherapy, *S* surgery, *C* chemotherapy

## Discussion

UPS is a rare and aggressive soft tissue sarcoma of unknown etiology that may involve the intracranial space [1–4]. Data on intracranial UPS is sparse given its rarity, and the evolving classification of UPS has posed challenges in understanding and treating this malignancy. Given the limited number of cases reported in the literature, our study aimed to consolidate existing knowledge on the management of UPS.

The demographic profile of the included patients in our study shows a mean age of 42.5 and standard deviation of 23.7 years, approximately 10 years younger than the onset of extracranial UPS [5, 22, 63]. This discrepancy may be attributed to the relative rarity of intracranial UPS and a higher incidence among children. Remarkably, about 23% of the cases in our review involved children, in contrast to a much lower incidence of extracranial UPS in children [64].

Of all variables studied, tumor location had the highest prognostic value. OS significantly differed by site, with extra-axial tumors (meninges/calvarium) demonstrating a median OS that was not reached, compared with 8 months for intra-axial/skull base lesions. This pattern may be due to anatomic feasibility of achieving adequate resection and radiation delivery, whereas intra-axial and skull base tumors can limit surgical margins and safe radiation dose delivery. These findings mirror other malignant lesions of the intracranial space, such as primary angiosarcomas, where complete resection was the strongest prognostic factor, regardless of size or intracranial invasion [65]. No other prognostic values, such as age, sex, and treatment modality were of significance when determining EFS and OS.

No significant differences in survival were observed between those receiving CRS and those receiving less than CRS, which may reflect the small sample size as opposed to a deviation from modern soft-tissue sarcoma treatment guidelines. Our finding of no survival difference mirrors a recent series of skull base soft tissue sarcomas by Habib et al., where FNCLCC grade and anatomic location determined tumor control as opposed to treatment modality. These patients routinely received multimodal therapy, consistent with modern management guidelines. Additionally, we found that skull base location was associated with worse survival in our cohort (*p* = 0.016), consistent with Habib et al.’s observation that extension into the skull base and cavernous sinus negatively impacted tumor control [66]. Although response to chemotherapy in soft tissue sarcomas is generally limited, CRS was used as a surrogate for aggressive treatment in our study to reflect efforts to improve local control in intracranial lesions where resection alone may be insufficient [67]. However, surgery with chemoradiation suggests a potential benefit compared to other treatment combinations. Patients treated with CRS had the longest median survival, while those treated with chemotherapy and surgery had the shortest. Outcomes for those who received radiation and surgery fell in between, which is consistent with the recognized role of radiotherapy in improving local control in soft tissue sarcomas [68]. Aggressive management when feasible may be supported, though more robust datasets are required to determine significance.

When outside the intracranial space, histologic grade guides treatment as opposed to anatomic site. FNCLCC Grade 3 sarcomas, such as UPS, are treated with wide resection, radiotherapy, and chemotherapy [6, 16, 17, 69]. However, achieving a complete surgical resection may be difficult when the lesion involves the intracranial space. Surgical resection was the predominant treatment modality in our cohort, as nearly all patients underwent this intervention. Indeed, the results of our systematic review indicated outcomes following treatment of intracranial UPS were generally poor. In this cohort of 48 patients, nearly half experienced at least one recurrence and ~ 42% died during follow-up, with median EFS and OS of 18 months and 24 months, respectively. These survival estimates are in line with prior intracranial UPS series (OS ~ 16–27 months), further demonstrating the aggressiveness of this disease [35, 60]. By contrast, extracranial UPS cohorts in the extremities or trunk report substantially lower recurrence (~ 14–26%) and higher long-term survival, with a 5-year OS of 60–72% and median survival approaching a decade [63, 70, 71]. The most plausible explanation for this outcome likely reflects the challenge of achieving large surgical resections in the intracranial space. These differences support the need for the development of effective adjunctive therapies to enhance control and survival in patients with intracranial UPS.

In this systematic review, 14.6% of patients had a history of radiotherapy to an intracranial lesion prior to developing their intracranial UPS. Despite the possible association between radiation exposure and developing UPS, radiotherapy has been shown to improve survival and is now recommended as part of the treatment paradigm for UPS, especially for deep lesions [14, 15, 63, 72]. In contrast, the use of chemotherapy is not as well established for UPS. There is evidence supporting the use of an anthracycline with or without ifosfamide as treatment adjuncts, however studies on this topic remain extracranial [10, 14, 73]. Chemotherapy did not significantly impact the survival of patients in this review; however, this could be confounded by the heterogeneity in chemotherapy regimens across included studies.

The high rate of recurrence observed demonstrates the need for novel therapies, and immunotherapy has emerged as a potential option in UPS. Immune checkpoint inhibitors demonstrate response rates of 20–40% in extracranial UPS [74, 75]. When combined with radiotherapy, response rates may reach 90%, likely reflecting differences in tumor microenvironment [76, 77]. Predictive markers such as PD-1/PD-L1 expression may increase during radiotherapy, supporting its adjuvant use [78, 79]. In fact, one study of refractory maxillary sinus UPS reported a complete response to treatment with ipilimumab and nivolumab combined with radiotherapy [80]. It remains unclear whether similar responses can be expected in intracranial UPS due to the lack of site-specific data, though efficacy has been reported in brain metastases of other cancers [81]. These findings warrant further exploration for intracranial UPS.

The most significant limitation of the present study is the small sample size and inability to run meaningful statistical analyses. Other limitations include the risk of selection bias inherent in systematic reviews. Importantly, the definition of UPS has evolved over time due to advancements in diagnostic techniques, increasing the chance of earlier studies misreporting this rare clinical entity. Additionally, the transition to classification based on FNCLCC grade introduces heterogeneity across UPS research, which may confound data from older studies. Moreover, the included studies were predominantly retrospective case reports or small case series, limiting the strength of this review’s overall conclusions. Additionally, the scarcity of information on treatment regimens of intracranial UPS poses challenges in drawing conclusions about their efficacy. Lastly, because the included literature consists of case reports and small case series, quantifying publication bias is not applicable [82]. The JBI appraisal demonstrated that although diagnosis, clinical information, and outcomes were well reported, many studies omitted adverse events. As a result, the potential for selective reporting of favorable outcomes may inflate the survival estimates. Because intracranial UPS cases with rapid decline or perioperative complications are underrepresented in the literature, the survival curves generated from this dataset likely overestimate true clinical outcomes. Despite these limitations, our study provides a comprehensive overview of the current state of knowledge regarding intracranial UPS. The lack of data emphasizes the importance of continued reporting and further study of this rare clinical entity.

## Conclusion

Intracranial undifferentiated pleomorphic sarcoma is a rare and aggressive tumor with outcomes worse than those of extracranial sites. In this systematic review, we found that tumor location was the strongest prognostic factor, as extra-axial tumors had the longest median survival. Additionally, surgical resection with adjuvant chemotherapy and radiation was associated with longer median survival compared to other treatment combinations, though not statistically significant. This study consolidates the existing literature into a concise review, though the small sample size prevents definitive treatment recommendations. Continued reporting of this lesion will be essential to refine treatment guidelines and improve outcomes for patients with intracranial UPS.

## Supplementary Information

Below is the link to the electronic supplementary material.

Supplementary Material 1 (DOCX 52.6 KB)

## Data Availability

All data supporting the findings of this study are available in the article and the supplementary material. Our dataset consists of previously published case reports and case series identified through our systematic review. A comprehensive list of included studies is provided in Supplementary Data 5.
