# Supplementary material for: Management of intracranial undifferentiated pleomorphic sarcoma: a systematic review
Source: Neurosurg Rev. 2026 Jan 6;49(1):104. doi: 10.1007/s10143-025-04018-7 (PMC12769503; doi:10.1007/s10143-025-04018-7)
Supplement: Supplementary file 1 — Supplementary Material 1 (DOCX 52.6 KB) [file 10143_2025_4018_MOESM1_ESM.docx]

**Supplementary Material 1**

PRISMA Checklist, Search Strategy, and Comprehensive List of Studies

**Article:** Management of intracranial undifferentiated pleomorphic sarcoma: a systematic review

**Journal:** Neurosurgical Review

Noah B. Drewes, BS^1^; Jeffrey Z. Nie, MD^1,2^; Rafal Chojak, MD^3^; Khizar Nandoliya, BA^3^; Rishi Jain, BA^3^; Harrsha Congivaram, BA^3^; Umme H. Faisal, MD^3^; Lara Koutah, MS^3^; Niyant Vora, MD^1^; Elek A. Wellman, MD^1^; Matthew W. Weber, MD^1^; Nathan J. Nordmann, MD^1^; Jeffrey W. Cozzens, MD^1^; Devin V. Amin, MD, PhD^1^; Jose A. Espinosa, MD^1^; Breck Jones, MD^1^; Bruce M. Frankel, MD^1^; Leslie Acakpo-Satchivi, MD, PhD^1,4^

^1^Department of Surgery, Division of Neurosurgery, Southern Illinois University School of Medicine, Springfield, Illinois, USA

^2^Department of Neurosurgery, Neurological Institute, Cleveland Clinic Foundation, Cleveland, Ohio, USA

^3^Department of Neurological Surgery, Feinberg School of Medicine, Northwestern University, Chicago, IL, USA

^4^Neurological Surgery, Springfield Clinic, Springfield, Illinois, United States of America

**Corresponding Author:** Rafal Chojak

**Email:** rafal.chojak@northwestern.edu

**Supplemental Data 1:** PRISMA Checklist

| **Section and Topic** | **Item #** | **Checklist item** | **Location where item is reported** |
| --- | --- | --- | --- |
| **TITLE** | | |  |
| Title | 1 | Identify the report as a systematic review. | 1 - Title |
| **ABSTRACT** | | |  |
| Abstract | 2 | See the PRISMA 2020 for Abstracts checklist. | 6 - Abstract |
| **INTRODUCTION** | | |  |
| Rationale | 3 | Describe the rationale for the review in the context of existing knowledge. | 7 - Introduction |
| Objectives | 4 | Provide an explicit statement of the objective(s) or question(s) the review addresses. | 7 - Introduction |
| **METHODS** | | |  |
| Eligibility criteria | 5 | Specify the inclusion and exclusion criteria for the review and how studies were grouped for the syntheses. | 8 - Methods |
| Information sources | 6 | Specify all databases, registers, websites, organisations, reference lists and other sources searched or consulted to identify studies. Specify the date when each source was last searched or consulted. | 7-8 - Methods |
| Search strategy | 7 | Present the full search strategies for all databases, registers and websites, including any filters and limits used. | 7 – Methods  Supplementary Data 2 |
| Selection process | 8 | Specify the methods used to decide whether a study met the inclusion criteria of the review, including how many reviewers screened each record and each report retrieved, whether they worked independently, and if applicable, details of automation tools used in the process. | 7-8 – Methods |
| Data collection process | 9 | Specify the methods used to collect data from reports, including how many reviewers collected data from each report, whether they worked independently, any processes for obtaining or confirming data from study investigators, and if applicable, details of automation tools used in the process. | 7-8 - Methods |
| Data items | 10a | List and define all outcomes for which data were sought. Specify whether all results that were compatible with each outcome domain in each study were sought (e.g. for all measures, time points, analyses), and if not, the methods used to decide which results to collect. | 7 - Methods |
|  | 10b | List and define all other variables for which data were sought (e.g. participant and intervention characteristics, funding sources). Describe any assumptions made about any missing or unclear information. | 7 - Methods |
| Study risk of bias assessment | 11 | Specify the methods used to assess risk of bias in the included studies, including details of the tool(s) used, how many reviewers assessed each study and whether they worked independently, and if applicable, details of automation tools used in the process. | 8 - Methods |
| Effect measures | 12 | Specify for each outcome the effect measure(s) (e.g. risk ratio, mean difference) used in the synthesis or presentation of results. | 8-9 - Methods |
| Synthesis methods | 13a | Describe the processes used to decide which studies were eligible for each synthesis (e.g. tabulating the study intervention characteristics and comparing against the planned groups for each synthesis (item #5)). | 7 - Methods |
|  | 13b | Describe any methods required to prepare the data for presentation or synthesis, such as handling of missing summary statistics, or data conversions. | 7 - Methods |
|  | 13c | Describe any methods used to tabulate or visually display results of individual studies and syntheses. | 7 - Methods |
|  | 13d | Describe any methods used to synthesize results and provide a rationale for the choice(s). If meta-analysis was performed, describe the model(s), method(s) to identify the presence and extent of statistical heterogeneity, and software package(s) used. | 7 - Methods |
|  | 13e | Describe any methods used to explore possible causes of heterogeneity among study results (e.g. subgroup analysis, meta-regression). | N/A |
|  | 13f | Describe any sensitivity analyses conducted to assess robustness of the synthesized results. | N/A |
| Reporting bias assessment | 14 | Describe any methods used to assess risk of bias due to missing results in a synthesis (arising from reporting biases). | N/A |
| Certainty assessment | 15 | Describe any methods used to assess certainty (or confidence) in the body of evidence for an outcome. | N/A |
| **RESULTS** | | |  |
| Study selection | 16a | Describe the results of the search and selection process, from the number of records identified in the search to the number of studies included in the review, ideally using a flow diagram. | 9 - Results |
|  | 16b | Cite studies that might appear to meet the inclusion criteria, but which were excluded, and explain why they were excluded. | 9 - Results |
| Study characteristics | 17 | Cite each included study and present its characteristics. | 9 – Results  Supplementary Table 5 |
| Risk of bias in studies | 18 | Present assessments of risk of bias for each included study. | 8 - Methods |
| Results of individual studies | 19 | For all outcomes, present, for each study: (a) summary statistics for each group (where appropriate) and (b) an effect estimate and its precision (e.g. confidence/credible interval), ideally using structured tables or plots. | 8 - Results |
| Results of syntheses | 20a | For each synthesis, briefly summarise the characteristics and risk of bias among contributing studies. | 8 - Results |
|  | 20b | Present results of all statistical syntheses conducted. If meta-analysis was done, present for each the summary estimate and its precision (e.g. confidence/credible interval) and measures of statistical heterogeneity. If comparing groups, describe the direction of the effect. | 11 - Results |
|  | 20c | Present results of all investigations of possible causes of heterogeneity among study results. | N/A |
|  | 20d | Present results of all sensitivity analyses conducted to assess the robustness of the synthesized results. | N/A |
| Reporting biases | 21 | Present assessments of risk of bias due to missing results (arising from reporting biases) for each synthesis assessed. | N/A |
| Certainty of evidence | 22 | Present assessments of certainty (or confidence) in the body of evidence for each outcome assessed. | N/A |
| **DISCUSSION** | | |  |
| Discussion | 23a | Provide a general interpretation of the results in the context of other evidence. | 9-11 - Discussion |
|  | 23b | Discuss any limitations of the evidence included in the review. | 9-11 - Discussion |
|  | 23c | Discuss any limitations of the review processes used. | 9-11 - Discussion |
|  | 23d | Discuss implications of the results for practice, policy, and future research. | 9 -11 - Discussion |
| **OTHER INFORMATION** | | |  |
| Registration and protocol | 24a | Provide registration information for the review, including register name and registration number, or state that the review was not registered. | 7 - Methods |
|  | 24b | Indicate where the review protocol can be accessed, or state that a protocol was not prepared. | 7 - Methods |
|  | 24c | Describe and explain any amendments to information provided at registration or in the protocol. | N/A |
| Support | 25 | Describe sources of financial or non-financial support for the review, and the role of the funders or sponsors in the review. | 15 - Statements and Declarations |
| Competing interests | 26 | Declare any competing interests of review authors. | 15 - Statements and Declarations |
| Availability of data, code and other materials | 27 | Report which of the following are publicly available and where they can be found: template data collection forms; data extracted from included studies; data used for all analyses; analytic code; any other materials used in the review. | 9 – Results  Supplementary Table 5 |

**Supplemental Data 2:** Search syntax for PubMed and Embase completed on August 8, 2025

| PubMed  266 articles | ("intracranial" AND "undifferentiated pleomorphic sarcoma") OR ("intracranial" AND "malignant fibrous histiocytoma") OR ("central nervous system" AND "undifferentiated pleomorphic sarcoma") OR ("central nervous system" AND "malignant fibrous histiocytoma") OR ("skull" AND "undifferentiated pleomorphic sarcoma") OR ("skull" AND "malignant fibrous histiocytoma") OR ("brain" AND "undifferentiated pleomorphic sarcoma") OR ("brain" AND "malignant fibrous histiocytoma") OR ("meninges" AND "undifferentiated pleomorphic sarcoma") OR ("meninges" AND "malignant fibrous histiocytoma") |
| --- | --- |

| Embase  535 articles | intracranial' AND ('undifferentiated pleomorphic sarcoma'/exp OR 'undifferentiated pleomorphic sarcoma') OR ('intracranial' AND ('malignant fibrous histiocytoma'/exp OR 'malignant fibrous histiocytoma')) OR (('central nervous system'/exp OR 'central nervous system') AND ('undifferentiated pleomorphic sarcoma'/exp OR 'undifferentiated pleomorphic sarcoma')) OR (('central nervous system'/exp OR 'central nervous system') AND ('malignant fibrous histiocytoma'/exp OR 'malignant fibrous histiocytoma')) OR (('skull'/exp OR 'skull') AND ('undifferentiated pleomorphic sarcoma'/exp OR 'undifferentiated pleomorphic sarcoma')) OR (('skull'/exp OR 'skull') AND ('malignant fibrous histiocytoma'/exp OR 'malignant fibrous histiocytoma')) OR (('brain'/exp OR 'brain') AND ('undifferentiated pleomorphic sarcoma'/exp OR 'undifferentiated pleomorphic sarcoma')) OR (('brain'/exp OR 'brain') AND ('malignant fibrous histiocytoma'/exp OR 'malignant fibrous histiocytoma')) OR (('meninges'/exp OR 'meninges') AND ('undifferentiated pleomorphic sarcoma'/exp OR 'undifferentiated pleomorphic sarcoma')) OR (('meninges'/exp OR 'meninges') AND ('malignant fibrous histiocytoma'/exp OR 'malignant fibrous histiocytoma')) |
| --- | --- |

**Supplemental Data 3:** JBI Critical Appraisal Summary for Included Case Reports

| **Study** | **Q1** | **Q2** | **Q3** | **Q4** | **Q5** | **Q6** | **Q7** | **Q8** |
| --- | --- | --- | --- | --- | --- | --- | --- | --- |
| Aghili et al | Yes | Yes | Yes | Yes | Yes | Yes | No | Yes |
| Akimoto et al | Yes | Yes | Yes | Yes | Yes | Yes | No | Yes |
| Altaf et al | Yes | Yes | Yes | Yes | Yes | Yes | No | Yes |
| Asahi et al | Yes | Yes | Yes | Yes | Yes | Yes | No | Yes |
| Barnes et al | Yes | Yes | Yes | Yes | Yes | Yes | No | Yes |
| Bauerle et al | Yes | Yes | Yes | Yes | Yes | Yes | Yes | Yes |
| Berry et al | Yes | Yes | Yes | Yes | Yes | Yes | Yes | Yes |
| Bora et al | Yes | Yes | Yes | Yes | Yes | Yes | No | Yes |
| Caltabiano et al | Yes | Yes | Yes | Yes | Yes | Yes | No | Yes |
| Chitale et al | Yes | Yes | Yes | Yes | Yes | Yes | No | Yes |
| Gonzalez-Vitale et al | Yes | Yes | Yes | Yes | Yes | Yes | Yes | Yes |
| Hamlat et al | Yes | Yes | Yes | Yes | Yes | No | No | Yes |
| Hari et al | Yes | Yes | Yes | Yes | Yes | Yes | Yes | Yes |
| Hatashita et al | Yes | Yes | Yes | Yes | Yes | Yes | No | Yes |
| Hayami et al | Yes | Yes | Yes | Yes | Yes | Yes | Yes | Yes |
| Joo et al | Yes | Yes | Yes | Yes | Yes | Yes | Yes | Yes |
| Kalyanaraman et al | Yes | Yes | Yes | Yes | Yes | Yes | Yes | Yes |
| Krishnamurthy | Yes | Yes | Yes | Yes | Yes | Yes | No | Yes |
| Kuzeyli et al | Yes | Yes | Yes | Yes | Yes | Yes | No | Yes |
| Martinez-Salazar | Yes | Yes | Yes | Yes | Yes | Yes | No | Yes |
| Matsuura et al | Yes | Yes | Yes | Yes | Yes | Yes | Yes | Yes |
| Mitsuhashi et al | Yes | Yes | Yes | Yes | Yes | Yes | Yes | Yes |
| Ozdemir et al | Yes | Yes | Yes | Yes | Yes | Yes | Yes | Yes |
| Ozercan et al | Yes | Yes | Yes | Yes | Yes | Yes | No | Yes |
| Ozhan et al | Yes | Yes | Yes | Yes | Yes | Yes | Yes | Yes |
| Pople et al | Yes | Yes | Yes | Yes | Yes | Yes | Yes | Yes |
| Roosen et al | Yes | Yes | Yes | Yes | Yes | Yes | No | Yes |
| Schiffer et al | Yes | Yes | Yes | Yes | Yes | Yes | No | Yes |
| Schmitt et al | Yes | Yes | Yes | Yes | Yes | Yes | Yes | Yes |
| Schrader et al | Yes | Yes | Yes | Yes | Yes | Yes | Yes | Yes |
| Sima et al | Yes | Yes | Yes | Yes | Yes | Yes | Yes | Yes |
| Simpson et al | Yes | Yes | Yes | Yes | Yes | Yes | Yes | Yes |
| Tsutsumi et al | Yes | Yes | Yes | Yes | Yes | Yes | No | Yes |
| Ueda et al | Yes | Yes | Yes | Yes | Yes | Yes | No | Yes |
| Wapshott et al | Yes | Yes | Yes | Yes | Yes | Yes | Yes | Yes |
| Yoshida et al | Yes | Yes | Yes | Yes | Yes | Yes | Yes | Yes |
| Zhang et al | Yes | Yes | Yes | Yes | Yes | Yes | No | Yes |
| Q1: Were the patient’s demographic characteristics clearly described?  Q2: Was the patient’s history clearly described and presented as a timeline?  Q3: Was the current clinical condition of the patient on presentation clearly described?  Q4: Were diagnostic tests or assessment methods and the results clearly described?  Q5: Was the intervention(s) or treatment procedure(s) clearly described?  Q6: Was the post-intervention clinical condition clearly described?  Q7: Were adverse events (harms) or unanticipated events identified and described?  Q8: Does the case report provide takeaway lessons? | | | | | | | | |

*JBI* Joanna Briggs Institute

**Supplemental Data 4:** JBI Critical Appraisal Summary for Included Case Series

| **Study** | **Q1** | **Q2** | **Q3** | **Q4** | **Q5** | **Q6** | **Q7** | **Q8** | **Q9** | **Q10** |
| --- | --- | --- | --- | --- | --- | --- | --- | --- | --- | --- |
| Alaggio et al | Yes | Yes | Yes | Unclear | Yes | Yes | Yes | Yes | Unclear | N/A |
| Oliveira et al | Yes | Yes | Yes | Unclear | Yes | Yes | Yes | Yes | Unclear | Yes |
| Peng et al | Yes | Yes | Yes | Unclear | Yes | Yes | Yes | Yes | Unclear | Yes |
| Q1. Were there clear criteria for inclusion in the case series?  Q2. Was the condition measured in a standard, reliable way for all participants included in the case series?  Q3. Were valid methods used for identification of the condition for all participants in the case series?  Q4. Did the case series have consecutive inclusion of participants?  Q5. Did the case series have complete inclusion of participants?  Q6. Was there clear reporting of the demographics of the participants in the study?  Q7. Was there clear reporting of clinical information of the participants?  Q8. Were the outcomes or follow-up results of cases clearly reported?  Q9. Was there clear reporting of the presenting sites’/clinics’ demographic information?  Q10. Was statistical analysis appropriate? | | | | | | | | | | |

*JBI* Joanna Briggs Institute

**Supplemental Data 5**: Comprehensive Review of Literature (n = 48)

| **Author** | **Age** | **Sex** | **Location** | **Tx** | **Presenting Symptoms** | **Recur** | **EFS** | **OS** |
| --- | --- | --- | --- | --- | --- | --- | --- | --- |
| Aghili et al | 57 | M | L temporal lobe | R/S | headache, nausea, decreased consciousness | - | 6 | 6* |
| Akimoto et al | 61 | M | L frontotemporal area | C/R/S | impaired spontaneous movement and word recall | + | 3 | 12 |
| Alaggio et al | 6 | M | - | C/R/S | - | + | - | 15 |
| Alaggio et al | 14 | F | Posterior fossa | C/R/S | - | + | - | 60* |
| Altaf et al | 25 | M | L frontal lobe | S | blurred vision, headaches | - | 30 | 30* |
| Asahi et al | 69 | F | L frontotemporal bone | S | painless growing scalp mass | + | 3 | 22 |
| Barnes et al | 67 | F | L sphenoid/cavernous sinus | R/S | headaches, blurred vision, L facial pain | - | 8 | 8* |
| Bauerle et al | 61 | M | R parietal bone | C/S | painless growing scalp mass | + | 3 | 4* |
| Berry et al | 75 | M | R temporal lobe, invading sphenoid ridge | R/S | headaches, blurred vision, personality changes | - | 6 | 6 |
| Bora et al | 5 | F | L parietal lobe | C/S | headaches, vomiting, fever, tonic-clonic seizures, R hemiplegia | + | 3 | 11 |
| Caltabiano et al | 71 | F | L parietal region | S | aphasia, reduced visual fields, R limb paresis | - | 6 | 6* |
| Chitale et al | 18 | M | L temporal bone | C/R/S | growing scalp mass | + | 2 | 14* |
| Gonzalez-Vitale et al | 37 | M | Sella turcica/cavernous sinus, invading hypothalamus | R | opthalmoplegia | - | 5 | 5 |
| Hamlat et al | 44 | F | R cerebellar hemisphere | S | headache, vomiting | + | 1 | 28* |
| Hari et al | 9 | F | L temporoparietal region | C/S | headache, nausea, vomiting, decreased vision | + | 1.5 | 8* |
| Hatashita et al | 45 | M | R frontal bone | R/S | scalp mass | - | 24 | 24* |
| Hayami et al | 21 | F | R temporal scalp in cranial bone defect following craniotomy, invading intracranially | S | scalp mass, seizure | + | 2 | 14* |
| Joo et al | 43 | F | R parietal bone | R/S | scalp mass | + | 3 | 3* |
| Kalyanaraman et al | 12 | M | L frontal/parietal area | C/R/S | R hemiplegia, aphasia | - | 18 | 18* |
| Krishnamurthy | 5 | F | R temporal bone | S | scalp mass | - | 12 | 12* |
| Kuzeyli et al | 58 | M | R temporoparietal region | R/S | headache | - | 6 | 6 |
| Martinez-Salazar | 13 | F | L frontal lobe, invading L frontal lobe | S | headaches, nausea, lethargy | - | 9 | 9* |
| Matsuura et al | 72 | M | L temporo-occipital bone | R/S | scalp mass | + | 5 | 5.5 |
| Mitsuhashi et al | 58 | F | R frontal lobe | S | L partial seizures | + | 1 | 7 |
| Oliveira et al | 11 | M | R frontal lobe | C/R/S | - | + | 18 | 26 |
| Oliveira et al | 17 | F | L frontal lobe | R/S | - | + | 11 | 24 |
| Oliveira et al | 63 | F | R parietal lobe | R/S | - | - | 3 | 3 |
| Ozdemir et al | 42 | M | L frontoparietal region | C/R/S | headache, speech impairment | + | 4 | 4* |
| Ozercan et al | 63 | M | Parietal region of calvarium, invading parietal bone | S | scalp bleeding | - | 11 | 11* |
| Ozhan et al | 5 | F | L parietal region | C/R/S | headache, vomiting | + | 2 | 2* |
| Peng et al | 44 | M | L parieto-occipital lobe | C/R/S | headache | - | 4 | 4 |
| Peng et al | 19 | M | R frontal lobe | C/R/S | headache | + | 1 | 5 |
| Peng et al | 34 | F | L temporal lobe | R/S | headache | - | 8 | 8 |
| Peng et al | 57 | F | R temporal lobe | R/S | headache | - | 6 | 6 |
| Peng et al | 69 | F | Anterior cranial fossa | R/S | visual deficiency | - | 4 | 4 |
| Peng et al | 50 | F | Posterior cranial fossa | C/R/S | headache | + | 24 | 24* |
| Pople et al | 5 | M | R frontal lobe | R/S | asymptomatic | + | 18 | 24* |
| Roosen et al | 45 | M | L thalamus | S | R paresthesias, hemiparesis, motor aphasia | + | 8 | 8 |
| Schiffer et al | 72 | F | L parietal region | R/S | R paresis, partial seizure | - | 6 | 6* |
| Schmitt et al | 59 | M | R pons in posterior fossa | C/S | headache, gait unsteadiness, blurry vision | - | 4 | 4 |
| Schrader et al | 45 | M | Dura overlying the petrous bone | R/S | impaired hearing, mixed aphasia | + | 6 | 6* |
| Sima et al | 65 | M | R frontal lobe | R/S | personality changes, memory impairment, confusion, lack of spontaneous speech, weakness, lethargy, difficulty walking | - | 7 | 7 |
| Simpson et al | 65 | M | R parietal/posterior frontal/deep temporal area | C/R/S | personality changes, drowsiness, headaches, L hemiparesis | - | 5 | 5* |
| Tsutsumi et al | 69 | F | Dura surrounding superior sagittal sinus/falx extending to the R | R/S | LLE numbness, hemiplegia | - | 27 | 27* |
| Ueda et al | 18 | F | Orbit with extension into skull base and dura at temporal tip | R/S | exophthalmos, double vision | - | 12 | 12* |
| Wapshott et al | 79 | M | Midline frontal bone, invading dura | R/S | scalp mass | - | 27 | 27* |
| Yoshida et al | 45 | F | L parietal bone, invading dura and parenchyma around central sulcus | C/R/S | scalp mass | - | 28 | 28* |
| Zhang et al | 51 | F | Cerebellar vermis | S | blurry vision, cerebellar ataxia | - | 6 | 6* |

O*S* Overall Survival, *EFS* event-free survival, *R* radiotherapy, *S* surgery, *C* chemotherapy, * = alive at time of review
